# The assessment of autonomic nervous function in patients with gastrointestinal malignancies and its relationship with clinical characteristics

**DOI:** 10.3389/fnins.2025.1542224

**Published:** 2025-02-28

**Authors:** Zhang Xiwen, Feng Qiyun, Li Chuqiao, Jiang Anqi, Wu Zhenzhen, Deng Qiong, Peng Yu, Wang Chunlin

**Affiliations:** ^1^Department of Oncology, Nanfang Hospital, Southern Medical University, Guangzhou, China; ^2^Department of Geriatrics, Zaozhuang Mental Health Center, Zaozhuang, Shandong, China; ^3^Department of Neurology, Nanfang Hospital, Southern Medical University, Guangzhou, China; ^4^Department of Neurology, Guangzhou First People’s Hospital, School of Medicine, South China University of Technology, Guangzhou, China

**Keywords:** gastrointestinal cancer, autonomic nervous function, COMPASS-31, CARTs, clinical characteristics

## Abstract

**Introduction:**

The gastrointestinal tract is the organ most extensively distributed by autonomic nerves, and researches have indicated a relationship between automatic nerves and the progression of gastrointestinal cancers. This study aimed to evaluate the autonomic nervous function in patients with gastrointestinal cancer and to explore its relationship with clinical characteristics.

**Methods:**

We employed the Composite Autonomic Symptom Score 31 (COMPASS-31) questionnaire and cardiovascular autonomic reflex tests (CARTs) to evaluate autonomic nervous function, while also conducting a thorough analysis of clinical data.

**Results:**

Our results showed that low white blood cell (WBC) count (OR = 0.461, 95% CI: 0.218–0.976, *p* = 0.043) and increased maximum tumor diameter (OR = 1.619, 95% CI: 1.025–2.555, *p* = 0.039) were risk factors for autonomic dysfunction according to the COMPASS-31 assessment. While hypertension (OR = 5.747, 95% CI: 1.186–27.862, *p* = 0.030) and elevated platelet-to-albumin ratio (PAR) (OR = 1.256, 95% CI: 1.025–1.540, *p* = 0.028) were identified as independent risk factors for autonomic dysfunction based on the CARTs results. Combining the findings from COMPASS-31 and CARTs revealed that older age (OR = 1.133, 95% CI: 1.015–1.264, *p* = 0.027) and vascular invasion (OR = 7.706, 95% CI: 1.391–42.684, *p* = 0.019) were also independent risk factors for autonomic dysfunction.

**Conclusion:**

Our findings reveal that these specific factors related to gastrointestinal cancers significantly influence autonomic nervous function. It is essential to evaluate autonomic nervous function and its associated risk factors in patients with gastrointestinal malignancies, which provide new insights into the intervention strategies for cancer diseases.

## Introduction

Gastrointestinal cancers, especially gastric and colorectal cancers, pose a significant threat to public health due to their high incidence and mortality rates (Arnold et al., 2020). The global lifetime risks of developing and dying from gastrointestinal cancers from birth to death are 8.20 and 6.17% in 2020 from a population-based systematic analysis (Wang et al., 2024). This situation requires a better understanding of their pathophysiology and risk factors related to the tumors’ formation (Keum and Giovannucci, 2019). Recent studies have highlighted the role of the autonomic nervous system (ANS) in cancer progression, particularly in gastrointestinal tumors (Hanoun et al., 2015; Silverman et al., 2021; Zahalka and Frenette, 2020). Cancer can drive neurogenesis, as well as nerves that may fuel GI tumor progression. The ANS, including sympathetic and parasympathetic nerves, mainly innervates the gastrointestinal tract, playing a crucial role in regulating various physiological functions and tumor microenvironments. For instance, a study has shown that alterations in the sympathetic and parasympathetic branches can influence tumor growth, metastasis, and patient outcomes in malignancies (Wang et al., 2024; Li et al., 2023). It is known that some neurotransmitters and neuropeptides, such as epinephrine and acetylcholine, drive the activation of various oncogenic pathways downstream of neural receptors within cancer cells (Wan et al., 2022). Despite increasing awareness of this relationship, there is still a lack of clinical data describing the status of autonomic nervous function in gastrointestinal cancer patients. It remains unclear whether autonomic dysfunction can predict cancer prognosis and serve as a marker for risk stratification. Additionally, it is uncertain which factors are associated with autonomic dysfunction in cancer patients and whether these factors can be targeted for personalized interventions. As autonomic dysfunction can increase the risk of sudden death and impact patient prognosis (Balcıoğlu and Müderrisoğlu, 2015; Goldberger et al., 2019), it is essential to focus on both the autonomic function of patients and its associated risk factors. Therefore, this study aims to evaluate autonomic nervous function in patients with gastrointestinal cancers, using COMPASS-31 and CARTs, both of which are reliable tools for assessing autonomic function and have shown good consistency (Peng et al., 2021). Additionally, this study will correlate autonomic function with clinical characteristics to uncover potential associations that may inform clinical practice. Our research aims to improve the understanding of the relationship between gastrointestinal cancers and the autonomic nervous system, which could lead to innovative therapies targeting tumors and neurogenesis.

## Method

### Study design

This is a retrospectively descriptive study.

### Study participants

A total of 55 newly diagnosed patients with gastrointestinal cancers (1 duodenal cancer, 1 jejunal cancer, and 53 colorectal cancer) at the Department of Oncology, Southern Hospital, Southern Medical University, during June 2022 to October 2023, were included. The exclusion criteria included: (1) patients who had already received chemotherapy or radiotherapy; (2) patients with diabetes, rheumatism or chronic heart disease; (3) abnormal nerve conduction study indicated the presence of prior peripheral neuropathy.

### Study tools

#### Composite autonomic symptom score 31 (COMPASS-31)

The COMPASS-31 is a self-assessment instrument published by the Mayo Clinic in 2012 and includes 31 items assessing six domains of autonomic function: orthostatic hypohemia, vasomotor function, secretion function, gastrointestinal function, bladder function, and pupillary motor function. The total score is 100 (Sletten et al., 2012). In our previous study, the cut-off value for Chinese people to judge abnormal autonomic nervous function was >20 (Peng et al., 2021), which was also applied in this study.

#### Cardiovascular autonomic reflex tests (CARTs)

Cardiovascular autonomic reflex tests (CARTs) are relatively objective tools for evaluating autonomic nervous function. CARTs include four tests: deep breathing, Valsalva maneuver, 30:15 heart rate ratio, and the decrease in systolic blood pressure after standing (Spallone et al., 2011). Each test is classified as normal, borderline, or abnormal, with scores of 0, 0.5, and 1, respectively. Therefore, the total score for CARTs ranges from 0.0 to 4.0, with nine possible grades. A score of ≥2.0 is classified as abnormal according to the American Diabetes Association (ADA) standard for diagnosing diabetic autonomic neuropathy (Spallone et al., 2011).

### Study processes

All participants were assessed for COMPASS and CARTs before receiving treatment. Then we collected clinical informations for all these participants, including: (1) demographic information such as gender, age, body mass index (BMI), history of hypertension, diabetes, smoking history, and Eastern Cooperative Oncology Group Performance Status (ECOG PS) score; (2) laboratory data including tumor markers, complete blood count (CBC), platelet/lymphocyte ratio (PLR), neutrophil/lymphocyte ratio (NLR), platelet/albumin ratio (PAR), C-reactive protein/albumin ratio (CAR), and systemic immune-inflammation index (SII); and (3) pathological data such as primary tumor site, degree of differentiation, tumor type, maximum diameter, regional lymph node metastasis, vascular invasion, and neural invasion. We divided these participants into normal or abnormal autonomic function groups according to the results of COMPASS-31 and CARTs, separately or combined. In the combined analysis, patients who scored abnormal on either COMPASS-31 or CARTs were classified into the abnormal group. After comparison between groups, univariate and multivariate regression analysis were performed to evaluate the risk factors of gastrointestinal cancer patients with abnormal autonomic nervous function.

### Study analysis

Statistical analysis was conducted using SPSS 26.0 software. Quantitative data with a normal distribution were presented as (*x* ± *s*), while count data were expressed as percentages (%). Intergroup comparisons (data not shown) were made using analysis of variance. Correlation analysis was performed using both Pearson and Spearman methods. Univariate and multivariate logistic regression analyses were used to identify relevant risk factors for autonomic nerve dysfunction and their association with cancer. A *p*-value of less than 0.05 was considered statistically significant.

### Ethic

This study was authorized and supervised by the Ethics Committee of Nanfang Hospital, Southern Medical University (NFEC-2022-362).

## Results

The study included 55 patients, consisting of 26 males and 29 females, with an average age of 53.4 ± 8.6 years. The average COMPASS-31 score was 14.3 ± 10.7, while the average CARTs score was 1.1 ± 0.8. The results indicated a significant positive correlation between COMPASS-31 and CARTs, with a correlation coefficient of *r* = 0.413 (*p* = 0.002).

### Analysis based on COMPASS-31 alone

According to whether the COMPASS-31 was >20, there were 40 cases in the normal group and 15 cases in the abnormal group. Univariate analysis showed that white blood cell (WBC) had statistical differences between groups, as shown in Table 1. Multivariate logistic regression analysis showed that decreased WBC and increased maximum tumor diameter were risk factors for autonomic dysfunction, as shown in Table 1.

**Table 1:** Factors influencing autonomic nervous function in patients with gastrointestinal malignancies according to the COMPASS-31.

	Normal group	Abnormal group	Univariate analysis		Multivariate analysis	
	*n* = 40	*n* = 15	OR (95% CI)	*P*	OR (95% CI)	*P*
Age, years	52.4 ± 9.0	56.2 ± 7.1	1.059 (0.979–1.145)	0.151		
Gender (male)	21 (52.5%)	5 (33.3%)	2.211 (0.640–7.639)	0.210		
Hypertension	7 (17.5%)	2 (13.3%)	0.725 (0.133–3.960)	0.711		
Neural invasion	17 (65.4%)	5 (50.0%)	0.529 (0.121–2.325)	0.400		
Vascular invasion	9 (33.3%)	5 (55.5%)	2.500 (0.536–11.651)	0.243		
Cancer stage						
II	4 (10.0%)	3 (20.0%)	N.A.			
III	19 (47.5%)	7 (46.7%)	0.491 (0.087–2.770)	0.421		
IV	17 (42.5%)	5 (33.3%)	0.392 (0.065–2.369)	0.308		
Tumor size, cm	4.2 ± 1.7	5.6 ± 2.0	1.469 (0.967–2.232)	0.071	1.619 (1.025–2.555)	**0.039**
CEA, U/mL	55.5 ± 178.6	35.5 ± 82.3	0.999 (0.994–1.004)	0.681		
WBC, ×10^9^/L	6.8 ± 2.6	5.2 ± 1.4	0.569 (0.352–0.920)	**0.021**	0.461 (0.218–0.976)	**0.043**
HGB, g/L	123.1 ± 20.0	113.0 ± 18.9	0.976 (0.948–1.005)	0.104		
ALB, g/L	42.6 ± 5.4	42.6 ± 3.4	1.001 (0.886–1.130)	0.991		
CRP, mg/L	15.0 ± 26.5	6.5 ± 14.9	0.978 (0.940–1.018)	0.281		
ALP, U/L	106.6 ± 71.1	73.6 ± 34.2	0.981 (0.957–1.006)	0.143		
PAR	7.3 ± 3.3	7.3 ± 2.9	0.993 (0.822–1.200)	0.941		

CEA, carcinoembryonic antigen; WBC, white blood cell; HGB, hemoglobin; ALB, albumin; CRP, C-reactive protein; ALP, alkaline phosphatase; PAR, platelet/albumin; OR, odds ratio; CI, credible interval; N.A., none application. Bold values indicate statistically differences, *p* < 0.05.

### Analysis based on CARTs alone

According to whether the CARTs were ≥2.0, there were 40 cases in the normal and 15 cases in the abnormal group (different from COMPASS-31). Univariate analysis showed that hypertension, Hemoglobin (HGB), albumin (ALB) and PAR had statistical differences between the groups, as shown in Table 2. Multivariate logistic regression analysis showed that hypertension and elevated PAR were risk factors for autonomic dysfunction, as shown in Table 2.

**Table 2:** Factors influencing autonomic nervous function in patients with gastrointestinal malignancies according to the CARTs.

	Normal group	Abnormal group	Univariate analysis		Multivariate analysis	
	*n* = 40	*n* = 15	OR (95% CI)	*P*	OR (95% CI)	*P*
Age, years	52.8 ± 8.8	56.9 ± 7.2	1.052 (0.952–1.163)	0.322		
Gender (male)	19 (47.5%)	7 (46.7%)	1.034 (0.315–3.396)	0.956		
Hypertension	4 (10.0%)	5 (33.3%)	4.500 (1.014–19.963)	**0.037**	5.747 (1.186–27.862)	**0.030**
Neural invasion	17 (63.0%)	5 (55.6%)	0.735 (0.159–3.393)	0.712		
Vascular invasion	8 (30.8%)	6 (60.0%)	3.375 (0.742–15.349)	0.140		
Cancer stage						
II	6 (15.0%)	1 (6.7%)	N.A.			
III	18 (45.0%)	8 (53.3%)	2.667 (0.274–25.939)	0.398		
IV	16 (40.0%)	6 (40.0%)	2.250 (0.222–22.795)	0.492		
Tumor size, cm	4.5 ± 2.0	4.7 ± 1.7	1.054 (0.788–1.055)	0.788		
CEA, U/mL	62.9 ± 181.2	15.7 ± 52.2	0.995 (0.985–1.006)	0.364		
LYM, ×10^9^/L	1.7 ± 0.4	1.4 ± 0.5	0.240 (0.054–1.060)	0.060		
HGB, g/L	124.1 ± 17.5	110.3 ± 23.5	0.967 (0.938–0.997)	**0.030**	0.976 (0.940–1.013)	0.205
ALB, g/L	43.4 ± 4.2	40.3 ± 6.1	0.872 (0.763–0.996)	**0.043**	0.948 (0.800–1.122)	0.534
AST, U/L	18.9 ± 9.6	29.2 ± 31.7	1.033 (0.985–1.083)	0.183		
PLR	179.2 ± 52.2	299.9 ± 266.5	1.006 (1.000–1.013)	0.062		
PAR	6.7 ± 1.8	8.9 ± 5.2	1.228 (1.005–1.500)	**0.045**	1.256 (1.025–1.540)	**0.028**

CEA, carcinoembryonic antigen; LYM, lymphocyte; HGB, hemoglobin; ALB, albumin; AST, aspartate transaminase; PLR, platelet/lymphocyte ratio; PAR, platelet/albumin ratio; OR, odds ratio; CI, credible interval; N.A., none application. Bold values indicate statistically differences, *p* < 0.05.

### Analysis of combined COMPASS-31 and CARTs

Either COMPASS-31 was >20, or CARTs were ≥2.0, there were 30 cases in the normal and 25 cases in the abnormal group. Univariate analysis showed that age, vascular invasion, lymphocyte (LYM), HGB had statistical differences between groups, as shown in Table 3. Multivariate logistic regression analysis showed that increased age and vascular invasion were independent risk factors for autonomic nervous dysfunction, as shown in Table 3.

**Table 3:** Factors influencing autonomic nervous function in patients with gastrointestinal malignancies according to the combined COMPASS-31 and CARTs.

	Normal group	Abnormal group	Univariate analysis		Multivariate analysis	
	*n* = 30	*n* = 25	OR (95% CI)	*P*	OR (95% CI)	*P*
Age, years	50.9 ± 9.1	56.4 ± 7.1	1.092 (1.011–1.179)	**0.026**	1.133 (1.015–1.264)	**0.027**
Gender	17 (56.7%)	9 (36.0%)	2.325 (0.782–6.915)	0.129		
Hypertension	4 (13.3%)	5 (20.0%)	1.625 (0.386–6.846)	0.508		
Neural invasion	14 (66.7%)	8 (53.3%)	0.571 (0.147–2.228)	0.420		
Vascular invasion	5 (23.8%)	9 (60.0%)	4.800 (1.137–20.272)	**0.033**	7.706 (1.391–42.684)	**0.019**
Cancer stage						
II	3 (10.0%)	4 (16.0%)	N.A.			
III	15 (50.0%)	11 (44.0%)	0.550 (0.102–2.972)	0.487		
IV	12 (40.0%)	10 (40.0%)	0.625 (0.112–3.477)	0.591		
Tumor size, cm	4.2 ± 1.7	5.0 ± 2.0	1.285 (0.877–1.882)	0.198		
CEA, U/mL	66.3 ± 202.8	30.5 ± 74.2	0.998 (0.994–1.003)	0.432		
LYM, ×10^9^/L	1.8 ± 0.4	1.4 ± 0.5	0.179 (0.044–0.727)	**0.016**		
HGB, g/L	126.2 ± 17.2	113.2 ± 21.0	0.964 (0.935–0.995)	**0.022**	0.980 (0.933–1.029)	0.423
ALB, g/L	43.7 ± 4.4	41.2 ± 5.2	0.895 (0.792–1.011)	0.073	0.459 (0.062–3.387)	0.445
UA, μmol/L	326.6 ± 99.8	274.5 ± 88.2	0.994 (0.988–1.000)	0.054		
AST, U/L	19.8 ± 10.8	24.0 ± 25.1	1.013 (0.980–1.048)	0.438		
PLR	170.8 ± 49.0	261.4 ± 212.0	1.008 (0.999–1.017)	0.082		

CEA, carcinoembryonic antigen; LYM, lymphocyte; HGB, hemoglobin; ALB, albumin; UA, uric acid; AST, aspartate transaminase; PLR, platelet/lymphocyte ratio; OR, odds ratio; CI, credible interval; N.A., none application. Bold values indicate statistically differences, *p* < 0.05.

## Discussion

This study investigated the relationship between autonomic nervous function and gastrointestinal cancers in diagnosed patients, using the COMPASS-31 questionnaire and CARTs to evaluate their autonomic function status. We identified correlations with several risk factors, including advanced age, larger maximum tumor diameter, hypertension, elevated PAR, reduced WBC count, and vascular invasion. This study highlighted the importance of examining patients’ autonomic function and the risk factors that may contribute to dysfunction of the autonomic nervous system.

This study offered new insights into how the autonomic nervous system (ANS) relates to gastrointestinal cancers, specifically focusing on assessing autonomic function in cancer patients. Previous research primarily examined the molecular mechanisms of the ANS in regulating different aspects of tumor development (Hajiasgharzadeh et al., 2020; March et al., 2020; Shao et al., 2016). The clinical evaluations of autonomic nervous function in cancer patients were uncommon. COMPASS-31 and CARTs are useful tools for evaluating autonomic function. COMPASS-31 is a questionnaire that is easy to use but relatively subjective; CARTs rely on electrophysiological devices and are more objective. They have shown good agreement in the diagnosis of diabetic autonomic neuropathy (Peng et al., 2021). Our study established a foundational approach for clinically evaluating autonomic nervous function in cancer patients, which paved the way for further prognostic research.

Our findings showed that certain clinical features—such as older age, hypertension, high PAR, low WBC count, increased maximum tumor diameter and vascular invasion—were associated with autonomic dysfunction in patients with gastrointestinal cancer. Autonomic decline is associated with age, and it has been confirmed in our previous study that COMPASS-31 score is positively correlated with age (Peng et al., 2021). At the same time, aging is also a chronic inflammatory state of the whole body, which can induce neuroinflammation, resulting in impaired autonomic nervous function (Li et al., 2023). Hypertension can both cause and result from abnormal autonomic nervous function. Clinical studies have confirmed the dysregulation of autonomic nervous control in the cardiovascular system, which is usually associated with increased sympathetic tone and decreased parasympathetic tone, which leads to a variety of cardiovascular diseases, including hypertension (Mancia and Grassi, 2014). Our findings also confirm the association between hypertension and autonomic disorders in patients with gastrointestinal cancer. Elevated PAR indicates increased platelet number, which is also an indicator of systemic inflammation. Thrombocytosis may be related to sympathetic nerve activation. Studies have reported that noradrenaline (NE) can induce megakaryocyte adhesion, migration and proplatelet formation, and promote the production of platelets (Chen et al., 2016). Similarly, adrenergic receptor agonists can induce vasoconstriction and local decrease in blood flow, triggering rapid calcium signaling of white blood cells and stopping cell movement, so sympathetic activation can impair the mobility of white blood cells (Devi et al., 2021; Globig et al., 2023), which can explain the leukopenia in the group of patients with autonomic dysfunction in this study. Therefore, decreased WBC and elevated platelets may be related to overactivation of sympathetic nerve, and are associated with poor prognosis of various tumors (Proctor et al., 2011). Larger tumor diameter and vascular invasion certainly reflect advanced tumor progression, which also supported the positive correlation between tumor development and autonomic nervous dysfunction. This also supported the previous findings that systemic immune responses, closely linked to autonomic regulation, might be weakened in cancer patients, affecting their prognosis (Globig et al., 2023; Saloman et al., 2016).

However, the present research is primarily a preliminary clinical study, and it is crucial to recognize its limitations. First, the small sample size restricts how widely the findings can be applied to larger patient populations. Currently, we are evaluating a larger group of patients with gastrointestinal cancer. Additionally, we aim to explore the relationships between autonomic nerve function and other types of cancer. Second, the present study lacks long-term follow-up data. Although we recognize that autonomic nervous dysfunction can result in a poor prognosis, it is essential to directly observe the outcome differences between the two patient groups. We are currently following up these groups of patients and observing changes in their survival curves. Finally, the study did not include laboratory experiments to confirm the link between the observed risk factors and autonomic dysfunction. This omission raises questions about the mechanistic insights derived solely from clinical data. Collectively, these limitations highlight the necessity for future studies to incorporate larger, more representative cohorts and robust experimental designs to substantiate the clinical observations reported herein.

## Conclusion

This investigation revealed important connections between autonomic nerve dysfunction and different clinical features in patients with gastrointestinal cancers. Several independent risk factors were identified, including older age, hypertension, high PAR, low WBC, increased maximum tumor diameter and vascular invasion. These findings enhanced our understanding of the relationship between autonomic dysfunction and cancer characteristics. Furthermore, these findings pave the way for future research on therapeutic interventions targeting autonomic pathways.

## Data Availability

The original contributions presented in the study are included in the article/supplementary material, further inquiries can be directed to the corresponding authors.
